# Dynamic Responses of Ground-Dwelling Invertebrate Communities to Disturbance in Forest Ecosystems

**DOI:** 10.3390/insects10030061

**Published:** 2019-02-26

**Authors:** Kayla I. Perry, Daniel A. Herms

**Affiliations:** 1 Department of Entomology, The Ohio State University, Ohio Agricultural Research and Development Center, 1680 Madison Ave., Wooster, OH 44691, USA; 2 The Davey Tree Expert Company, 1500 Mantua Street, Kent, OH 44240, USA; dan.herms@davey.com

**Keywords:** arthropods, canopy gap formation, emerald ash borer, exotic species, tornado, salvage logging, wind, woody debris

## Abstract

In forest ecosystems, natural and anthropogenic disturbances alter canopy structure, understory vegetation, amount of woody debris, and the properties of litter and soil layers. The magnitude of these environmental changes is context-dependent and determined by the properties of the disturbance, such as the frequency, intensity, duration, and extent. Therefore, disturbances can dynamically impact forest communities over time, including populations of ground-dwelling invertebrates that regulate key ecosystem processes. We propose conceptual models that describe the dynamic temporal effects of canopy gap formation and coarse woody debris accumulation following disturbances caused by invasive insects, wind, and salvage logging, and their impacts on ground-dwelling invertebrate communities. Within this framework, predictions are generated, literature on ground-dwelling invertebrate communities is synthesized, and pertinent knowledge gaps identified.

## 1. Introduction

Disturbances are relatively discrete events in time and space that increase heterogeneity at varying spatial scales [1,2,3], shaping long-term fluctuations in community dynamics and ecosystem processes [4,5,6]. These events can be characterized by their properties, with each disturbance differing in type, intensity, frequency, severity, extent, and duration [3,7,8]. These properties, along with the causal agent, characterize the disturbance regime [1] and determine the effect size of the disturbance event on the structure and function of ecosystems.

Natural disturbance regimes are integral to the maintenance of local complexity and landscape heterogeneity through the creation and spatial arrangement of biological legacies, which are “organisms, organically derived structures, and organically produced patterns” that remain in the disturbed patch [9,10]. Biological legacies include, but are not limited to, living residual trees, snags, newly downed boles and existing woody debris, tip-up mounds and pits from fallen trees, intact understory vegetation, advanced regeneration, and patches of undisturbed forest [2,11,12]. These structural features influence the rate and successional pathways of forest recovery following a disturbance event.

Anthropogenic pressures have become widespread. In many cases, they have different properties than natural disturbances with the potential to create novel environmental conditions that could lead to alternative stable states, and thus altered patterns of ecosystem structure and function [7]. Anthropogenic perturbations, alone or in combination with natural disturbances, may reduce or deplete biological legacies in the landscape [10], altering or exacerbating the effects on community and ecosystem dynamics [13]. Therefore, ecosystem responses to anthropogenic disturbances likely will be site-specific [8,14] and dependent on multiple interacting factors.

## 2. Disturbance in Forest Ecosystems

In forest ecosystems, disturbances shape stand structure and function by redistributing resources on multiple spatial scales through reductions in living biomass, changes in nutrient cycling, and altered successional trajectories [2], thereby creating a mosaic of dynamic habitat patches that vary spatially and temporally over the landscape [3,15]. These events can range from small-scale, low intensity, frequent events affecting individual trees to large-scale, high intensity, infrequent events affecting entire stands [1]. Disturbances impact forest communities directly through individual tree mortality and indirectly by changing resource availability, habitat structure, competitive interactions, and ecosystem processes [16,17]. Depending on the disturbance properties, the effects on communities can have major economic and ecological implications [18,19,20].

Responses of forest communities, such as ground-dwelling invertebrates, to natural and anthropogenic disturbances likely will depend on species-specific life history strategies and evolutionary history with the disturbance regime. Ground-dwelling invertebrates are integral to forest ecosystems due to their high taxonomic and functional diversity and contribution to ecosystem services [21,22,23]. Life history traits, such as physiological tolerances, habitat preferences, and dispersal capacity influence the responses of invertebrates to natural disturbances and their ability to adapt to novel perturbations [15,24,25]. Most ground-dwelling invertebrates are thought to be relatively immobile because of their small size and limited dispersal capacity [26,27], which has implications for recolonization following disturbance in forests.

In eastern North American forests, invasive insects and wind storms are significant causes of tree mortality [1,4,28,29]; globally these disturbances affect millions of hectares [30]. Establishment and spread of invasive insects are becoming more frequent worldwide and represent a major threat to biodiversity and natural habitats [31,32,33] because they can modify or create new disturbances with potentially novel combinations of properties [34,35]. Wind is a dominant natural disturbance agent affecting forests in eastern North America [36], with the intensity and frequency of strong, stand-replacing storms predicted to increase with climate change [37,38]. As disturbances that cause tree mortality become more frequent and widespread, land managers may face increased pressures to salvage timber to recover economic losses with implications for conservation objectives and retention of biological legacies in the landscape.

Formation of canopy gaps of varying sizes is a common consequence of disturbance agents such as invasive insects and wind that cause tree mortality in forests. Canopy gaps alter the forest floor environment by increasing light availability, altering soil temperature and moisture regimes, increasing soil disturbance, stimulating understory vegetation regeneration and growth, decreasing leaf litter moisture and depth, and increasing volumes of downed fine woody debris (FWD; <10 cm in diameter at the large end) and coarse woody debris (CWD; ≥10 cm in diameter at the large end) [39,40,41,42,43,44,45,46,47]. Canopy openings also alter the abiotic environmental variation, including the range in daily minimum and maximum temperatures and moisture levels of leaf litter and soil [42,46,48]. Local (e.g., size, shape, orientation, structure, and amount of edge) and landscape (e.g., gap isolation, number of gaps, and forest structure) characteristics can affect the extent of environmental differences on the forest floor between the gap and surrounding undisturbed forest [49].

Canopy gaps and their associated environmental changes affect the abundance and diversity of ground-dwelling invertebrates, including insects and spiders that are key regulators of ecosystem processes [40,50,51,52,53,54]. For example, changes in leaf litter and soil moisture regimes altered the abundances and distributions of Gastropoda [55], Acari [56], Araneae [57], Collembola [56,58,59,60,61], Gryllidae [58], Carabidae [62,63], and Formicidae [56,58]. Responses of invertebrates to natural and anthropogenic disturbances have implications for ecosystem services, including decomposition, nutrient cycling, and maintenance of soil structure [21,22,64].

Tree mortality caused by invasive insects and wind also leads to the accumulation of logs and large branches (CWD; coarse woody debris) on the forest floor as trees fall. Downed CWD is a fundamental structural component (i.e., biological legacy) that increases habitat complexity [65] and provides resources for flora and fauna, including food, habitat, and sites for sprouting, breeding, and overwintering [66,67,68]. As CWD decays over time, the communities utilizing this resource change along with the physical and chemical properties of the wood [67,69,70]. For non-xylophagous invertebrates, CWD primarily regulates abiotic conditions at the soil surface during the early stages of decay when the bark is still firmly attached [67,71,72], whereas food and habitat become abundant after fungal colonization and insect tunneling as decay progresses [67,69]. Because wood decomposition occurs on timescales of 50–200 years [67,69,73], the effects of CWD on ground-dwelling invertebrate communities have the potential to be long-lasting.

The differential effects of canopy gap formation and accumulation of CWD on the forest floor environment likely will generate different responses in ground-dwelling invertebrates. However, these factors are inextricably linked, which make isolating their individual effects after natural and anthropogenic disturbances a challenge. Context dependent patterns of disturbances may cause one of these factors to contribute more to the structure and function of ground-dwelling invertebrate communities. Moreover, interactions between canopy gaps and CWD may result in unexpected responses over time.

## 3. Responses of Ground-Dwelling Invertebrates to Patterns of Forest Disturbance

Disturbances that cause tree mortality produce dynamic temporal and spatial patterns of canopy gap formation and CWD accumulation in forest ecosystems. Thus, their effects on ground-dwelling invertebrates also will be dynamic and dependent on the properties of the disturbance. Monitoring ground-dwelling invertebrates is ideal for detecting and characterizing forest responses to natural and anthropogenic disturbances [74] because they respond quickly to changes in habitat complexity and microclimate on the forest floor, such as soil moisture [58], coarse woody debris [75], leaf litter [76], and vegetation cover [52]. Moreover, Araneae, Collembola, Carabidae, and Formicidae have been used as indicators of environmental change [77,78,79,80].

The impacts of natural and anthropogenic disturbance on ground-dwelling invertebrate communities have been investigated extensively. Herein, we propose three conceptual models that make testable predictions regarding the dynamic temporal effects of canopy gap formation and coarse woody debris accumulation in response to disturbances caused by invasive insects, wind, and salvage logging, and their impacts on ground-dwelling invertebrate communities (Table S1). Our goals in the development of these models were: (1) to provide a framework for synthesizing the published literature; and (2) to generate testable predictions that may inform future research where knowledge gaps exist.

### 3.1. Invasive Insects

In forests of eastern North America, most tree species exist in a highly diverse and mixed forest community. Patterns of tree mortality caused by native insects, such as wood-boring beetles in the families Buprestidae and Cerambycidae, cause small gaps that are unevenly distributed throughout the landscape because these species typically attack stressed and dying trees [81,82]. Gap-phase dynamics caused by these native insects are similar in size and frequency to those caused by the natural senescence of old trees. However, there are exceptions. Native insects such as spruce budworm (*Choristoneura fumiferana* (Clem.)) [83,84] and forest tent caterpillar (*Malacosoma disstria* Hbn.) [85,86] can cause large-scale tree mortality during outbreak years, and some Scolytinae can overwhelm healthy trees with aggregation behaviors [87,88,89].

Extensive tree mortality caused by invasive insect species, such as gypsy moth (*Lymantria dispar* L.), hemlock woolly adelgid (*Adelges tsugae* Annand), beech scale (*Cryptococcus fagisuga* Lind.), and emerald ash borer (*Agrilus planipennis* Fairmaire), results in a different spatial pattern of forest gaps than does isolated tree mortality [35]. Emerald ash borer (EAB) is an invasive wood-borer (Coleoptera: Buprestidae) that has killed hundreds of millions of ash trees (*Fraxinus* spp.) since it was accidentally introduced into eastern North America from southeast Asia [90]. The range of EAB is increasing rapidly, and because *Fraxinus* is one of the most widely distributed tree genera in North America, the scale of impact will become continental. Consequently, EAB is causing widespread, nearly simultaneous formation of canopy gaps [91,92], with significant ecological and economic impacts [35,93,94,95]. Individual gaps from EAB-induced ash mortality are relatively small because most ash species exist in diverse forest communities. The speed, synchrony, and specificity of EAB-induced ash mortality make it a unique phenomenon in eastern North America [91,96,97], and warrant a focus on the indirect effects of ash mortality on ground-dwelling invertebrate communities.

**Figure 1:**
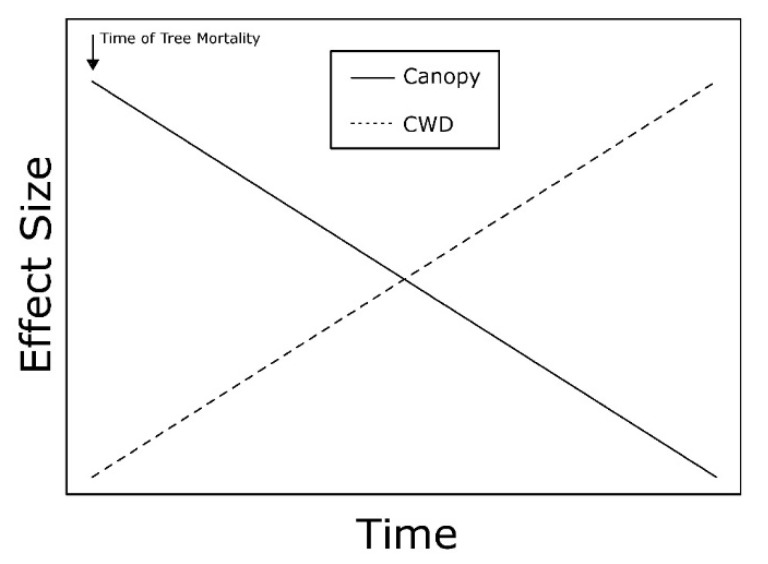
Conceptual model depicting the predicted temporal relationship between the effect sizes of canopy gaps and accumulation of coarse woody debris (CWD) caused by emerald ash borer-induced ash mortality on ground-dwelling invertebrate communities. Time of tree mortality denotes when ash trees die, and not the time of emerald ash borer infestation.

Ash mortality caused by EAB increases the frequency of gaps in the canopy and ash CWD on the forest floor [35,91,97]. For invasive insects, such as EAB, an inverse temporal relationship is predicted for the effect sizes of canopy gaps and accumulation of CWD on ground-dwelling invertebrate communities (Figure 1). As the effects of gaps diminish with canopy closure, the effects of CWD increase over time as trees fall [50,98,99]. These patterns have the potential to impact populations of ground-dwelling invertebrates in dynamic ways.

Based on this framework, canopy gaps are predicted to have the greatest effects on ground-dwelling invertebrates during early stages of EAB-induced ash mortality. Once characteristic signs and symptoms of EAB infestation become visible, such as D-shaped exit holes from adults and canopy decline, ash trees typically die within 2–4 years [90,100]. Over time as EAB populations build, ash mortality increases rapidly, and Klooster et al. [94] reported that more than 50% of trees in a stand died within a four-year period. Canopy gaps are presumably at their maximum size soon after tree death. Average sizes of gaps during early stages of ash mortality ranged from 18.8% (±1.8) to 26.5% (±2.0) canopy openness [40,92]. Canopy gaps altered the forest floor environment by increasing light availability, soil temperature, and soil moisture [40].

Vertical growth of suppressed understory trees and lateral growth of surrounding dominant and codominant canopy trees close the gaps following the increase in space and resource availability from ash mortality. Because individual gaps tend to be relatively small, in some cases ranging from 1–4 dead ash trees [40,50], substantial canopy closure can occur within 4–8 years. During late stages of EAB-induced ash mortality, canopy gaps ranged in size from 1–10% canopy openness [98,101]. Site-specific conditions likely will interact with ash density and spatial distribution to determine time until canopy closure. 

Our model predicts that ash CWD will have the greatest effects on ground-dwelling invertebrates during late stages of EAB-induced ash mortality, as this accumulation of woody debris coincides with canopy closure. During early stages of ash mortality, trees can remain standing as snags for several years, resulting in minimal effects on invertebrates because little downed CWD has accumulated above background levels. Long [102] observed an average of 2.2% cover of downed CWD in forests experiencing early stages of EAB-induced ash mortality. Ash snags fall over time as they uproot or snap along the bole. Near the epicenter of the EAB invasion in southeastern Michigan, USA, ash fell at a rate of 3.5% per year once the stand had reached 90% ash mortality [97]. Large quantities of ash CWD can accumulate on the forest floor depending on the density of ash [96,97], substantially more than in forests that are not affected by EAB. The average percentage cover of downed ash CWD was 19.3% (±0.8) in forests experiencing late stages of ash mortality [98]. The effects of downed ash CWD are predicted to persist for many years as decomposition slowly occurs, although *Fraxinus* wood is less resistant to decay [73].

Studies investigating the impacts of ash mortality caused by EAB on ground-dwelling invertebrates support the predicted effects of canopy gaps and CWD, but effects of tree mortality caused by other invasive insects were variable. During early stages of ash mortality, when canopy gaps are presumably at their maximum size and levels of downed CWD are low, Carabidae assemblages were altered by the formation of gaps, but not accumulation of CWD [40,92]. Gandhi et al. [92] documented initial decreases in Carabidae activity-abundance and diversity, as well as changes in species composition as canopy openness increased. Perry and Herms [40] also reported initial decreases in Carabidae activity-abundance and changes in species composition in canopy gaps created by EAB. However, in both studies, effects on Carabidae assemblages were ephemeral, and canopy gaps had no effect during the second year. Findings from Gandhi et al. [92] and Perry and Herms [40] suggest canopy gaps have the greatest impact on Carabidae during early stages of EAB-induced ash mortality, but assemblages may be resilient to this degree of canopy disturbance.

Perry and Herms [50] reported decreased ground-dwelling invertebrate richness and diversity in canopy gaps created by EAB-induced ash mortality. Activity-abundances of Opiliones, Carabidae, Scarabaeidae, Rhaphidophoridae, and three families of Collembola (Hypogastruridae, Isotomidae, and Sminthuridae) also decreased in canopy gaps. Diplopoda in the Order Polydesmida were the only invertebrates that increased in activity-abundance in gaps. Although low levels of CWD had minimal effects on most ground-dwelling invertebrates, activity-abundances of Isopoda and Gastropoda increased near ash CWD [50].

During late stages of ash mortality when ash CWD accumulates on the forest floor as canopy gaps close, Ulyshen et al. [101] documented higher densities of ground-dwelling invertebrates near ash logs, but minimal effects of small gaps. Annelida, Araneae, Opiliones, Isopoda, Diplopoda, Coleoptera, and Collembola were more abundant in leaf litter near ash logs than away [101]. Perry and Herms [98] further investigated late stages of ash mortality on ground-dwelling invertebrates in the same forest plots as Ulyshen et al. [101], and documented increased total activity-abundance, evenness, and diversity near recently fallen ash logs compared to more decayed logs. Decay class of CWD interacted with soil edaphic conditions to influence the structure of invertebrate communities during late stages of ash mortality [98].

Canopy gaps created by early stages of eastern hemlock (*Tsuga canadensis* (L.)) mortality from hemlock woolly adelgid (HWA) increased Formicidae activity-abundance and species richness [103], supporting the model predictions. Experimentally girdled hemlock to emulate gaps created by early stages of HWA-induced tree mortality [104] altered the composition of Coleoptera and Araneae assemblages compared to undisturbed hemlock stands [105] and increased the species evenness and diversity of Formicidae assemblages [106]. Although tree mortality and the formation of canopy gaps occurred within two years after girdling, hemlock snags were still standing after four years [104,107]. Increased volume of downed CWD has been reported in forest stands with high tree mortality caused by HWA [108], but to our knowledge, the effects of late stages of hemlock mortality on invertebrates have not been investigated. HWA-induced hemlock mortality typically occurs within 4–10 years [109,110,111], or longer, depending on initial tree health and HWA population growth rates. Decline and mortality of hemlock tends to occur more slowly than EAB-induced ash mortality, which may result in distinct disturbance properties.

Canopy gaps created by American beech (*Fagus grandifolia* (Ehrh.)) decline from beech bark disease (a disease complex of beech scale and an Ascomycete fungal species (*Nectria* spp.)) yielded no clear relationship between disease severity and ground-dwelling invertebrate richness or evenness [112]. However, insufficient information on the size of gaps created by beech mortality limited the interpretation of these results in the model framework. Most trees evaluated were reported as healthy [112], suggesting these forest stands were experiencing early stages of decline from beech bark disease.

### 3.2. Wind

Canopy gaps can be formed naturally by wind [36,113,114,115] from the death of a few trees by windthrow (tens of meters or less), or many trees (thousands of hectares) by intense windstorms, such as tornados, downbursts, derechos, and hurricanes [54,115]. Extensive tree mortality caused by wind can substantially alter the canopy, understory, and soil layers in forests.

**Figure 2:**
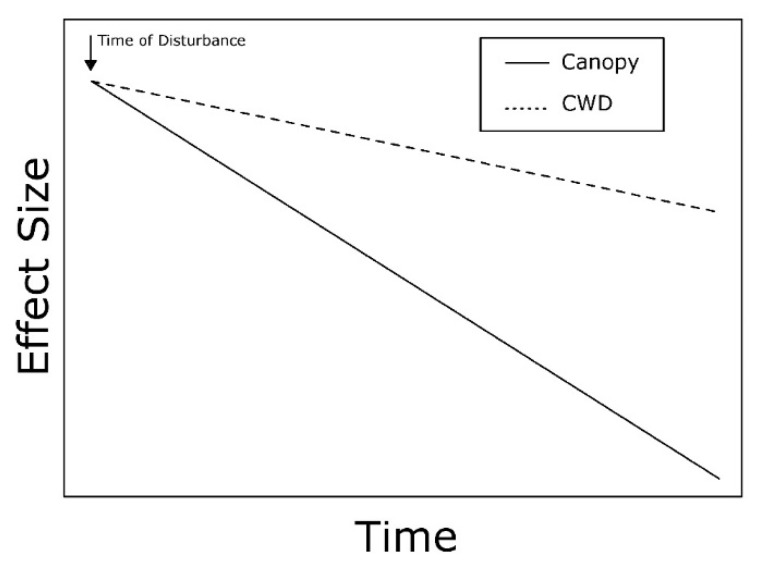
Conceptual model depicting the predicted temporal relationship between the effect sizes of canopy gaps and accumulation of coarse woody debris (CWD) caused by wind disturbance on ground-dwelling invertebrate communities. Time of disturbance denotes the windthrow event.

Wind disturbance causes the simultaneous formation of canopy gaps and accumulation of CWD when trees are uprooted. A negative temporal relationship is predicted for the effect sizes of canopy gaps and accumulation of CWD on ground-dwelling invertebrate communities (Figure 2). Therefore, canopy gaps and CWD are predicted to have the greatest effects on invertebrates immediately after the wind event, and then these effects decrease over time at different rates as gaps close and CWD decomposes.

Large, patchy gaps are formed from severe storms when many dominant and codominant canopy trees uproot, either directly from wind or indirectly from being in the path of treefall, and some remain standing, losing only branches. In contrast to canopy gaps created by EAB-induced ash mortality, which are smaller in size and spread across the landscape in a wave pattern, the distribution of gaps created by wind storms is stochastic and these gaps are often larger in size. Increased light availability in large gaps created by wind stimulates extensive growth of understory vegetation and advanced regeneration [116], which can form a dense “canopy” for ground-dwelling invertebrates.

Topography is altered when trees are uprooted, mixing organic and mineral soil layers, exposing rocks and roots, and forming pits and mounds on the soil surface [117,118,119]. Formation of pit-and-mound topography alters physical and chemical soil properties at the base of uprooted trees, forming distinct microsites that differ from undisturbed soil [119,120,121]. This fine-scale heterogeneity in microhabitats and soil properties may provide additional refugia, breeding, and overwintering sites for ground-dwelling invertebrates.

Trees fall to the ground nearly simultaneously and in the same direction when they uproot or snap from wind, producing a unique pattern of different species, types, and sizes of CWD accumulation [113,122]. This rapid accumulation of CWD results in a shift in carbon storage from live standing biomass to dead woody biomass on the forest floor [123]. The likelihood that a tree will fall is influenced by abiotic site factors (e.g., local topography and soil properties) and species-specific characteristics (e.g., size, root and canopy structure, wood strength, and prior insect or disease infestation) [2,113,124]. Trees generally have dense foliage when they fall and can remain alive for several years [13,122,125]. Trees that remain standing in the disturbed patch or along the forest edge tend to be more susceptible to future wind events [126]. 

Tree mortality caused by wind is predicted to have a negative temporal relationship in the effect sizes of canopy gaps and accumulation of CWD in forest ecosystems (Figure 2). Based on this model, canopy gaps and CWD have the greatest impacts on ground-dwelling invertebrates soon after wind disturbance and decrease over time at different rates. Effects of canopy gaps on ground-dwelling invertebrates are predicted to dissipate more quickly, especially if the impacts are mitigated by the rapid growth of understory vegetation, saplings, and advanced regeneration. In comparison, effects of CWD may be long-lasting because decomposition occurs on timescales of decades or longer [67]. Studies investigating the impacts of wind on ground-dwelling invertebrate communities generally report impacts (positive and negative) of canopy gaps, but few significant effects of downed CWD.

Lower ground-dwelling invertebrate diversity and biomass in canopy gaps (30 × 30 m plots), but no effects of woody debris, were reported following a manipulative experiment designed to emulate disturbance caused by hurricanes [41]. Large predators and detritivores were the primary taxa driving these patterns, while invertebrates that consume fungi, such as Acari, Collembola, and Psocoptera, increased in gaps [41]. Tree limbs and stems were added to the forest floor for the woody debris treatment, but not large boles, which suggests the effects of FWD were tested rather than CWD. Further, canopy gap size declined quickly from 15–20% canopy openness following canopy trimming to nearly 5% in 18 months [41].

Ground-dwelling invertebrate activity-abundance and biomass decreased in canopy gaps (0.1–1.5 ha) created by Hurricane Opal compared to nearby undisturbed forest [39]. Similar to patterns observed by Richardson et al. [41], these community responses were driven by decreased activity-abundances of dominant taxa representing multiple functional groups, including Carabidae, Araneae, Julida, Spirobolida, and Scolopendromorpha [39]. Percentage cover of downed CWD was higher in windthrow gaps, but contrary to the model predictions, a strong effect was not observed.

Perry [127] found ground-dwelling invertebrate activity-abundance was higher one year after a tornado, but was similar to nearby undisturbed forest two- and three-years post-disturbance. Invertebrate diversity was lower one year after the tornado, was higher during year two, and similar to undisturbed forest by year three [127]. Initial changes resulted in distinct community composition between windthrow gaps and undisturbed forest, but this difference also disappeared by year three. Activity-abundances of Spirobolidae, Araneae, Opiliones, Formicidae, Carabidae, Ptiliidae, and Staphylinidae were higher, while Scolopendromorpha, Julidae, Geotrupidae, and Aphodiinae were lower in windthrow gaps. High volume of downed CWD and increased growth of understory vegetation characterized windthrow gaps, which seemed to support forest and colonizing open-habitat species. These findings are consistent with model predictions that canopy gap formation and CWD accumulation will be greatest soon after wind disturbance.

Studies that investigated the effects of canopy gaps caused by wind disturbance on Formicidae assemblages found variable responses, but did not consider the accumulation of CWD [128,129,130]. Patrick et al. [128] surveyed Formicidae assemblages in canopy gaps (80 to 100 m^2^ in size) along a 1–12 year chronosequence thought to have been created by wind storms. Formicidae species richness was higher in canopy gaps (due to the presence of rare species) and positively correlated with insolation, but density and species composition were largely unaffected [128]. This indicates that the largest canopy gaps, which are presumably recent in the chronosequence, supported the most species and had the greatest impacts on Formicidae assemblages. Conversely, Feener Jr. and Schupp [130] found no difference in Formicidae activity-abundance or species richness between undisturbed forest and 1–2 year-old large canopy gaps (ca. 100 m^2^). Perhaps Formicidae, unlike many other taxa, respond positively to changes in microclimate, plant productivity, and other resources in canopy gaps, and thus spend more time foraging in these habitats [129,130].

Impacts of wind disturbance on Carabidae assemblages were largely consistent across studies, although gap size and downed CWD cover are often not reported between disturbed and undisturbed sites. Higher species richness and (or) diversity and altered composition of Carabidae assemblages were documented in forests affected by tornados [131,132] and hurricanes [133,134]. Assemblages in wind-disturbed forests were characterized by smaller, macropterous beetles, such as *Amara* and *Harpalus* species that prefer open, dry habitats and are more omnivorous, with seeds comprising a major portion of their diet. In comparison, nearby undisturbed forests were characterized by larger, brachypterous species that breed in the autumn, prefer moist habitats, and are primarily predaceous. Sklodowski and Garbalinska [131] sampled Carabidae six years following a tornado and found no signs that assemblages had recovered to a pre-disturbance state, suggesting these impacts can be long-lasting and more long-term studies are needed to document the recovery process. These findings also suggest that windstorms increase regional Carabidae species diversity in forest landscapes for years following the disturbance event. 

### 3.3. Salvage Logging

Salvage logging, or post-disturbance logging, removes commercially valuable standing and downed damaged, dying, and dead trees, as well as undamaged living trees from forest stands following natural disturbance [10,11,135]. Harvesting of timber after large-scale, stand replacing natural disturbances is a common management practice used to recover economic losses before trees deteriorate [136], and in response to the perceived risk of future insect or disease outbreaks and fire [11,113,137]. Depending on management objectives, harvesting may also be used to ensure safe access to public land [13,125,138]. Effects of salvage logging will vary based on a variety of factors including the harvesting method [11,139], but also site-specific conditions, such as soil type and moisture levels [139,140].

**Figure 3:**
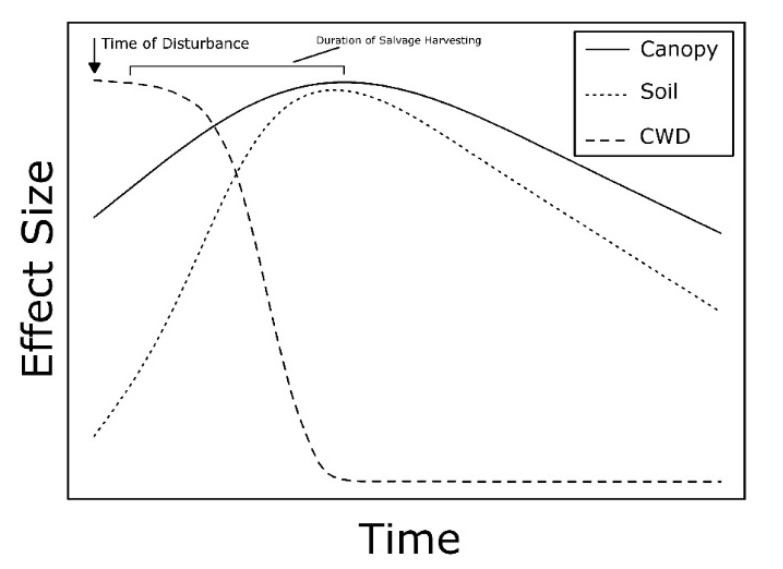
Conceptual model depicting the predicted temporal relationship between the effect sizes of canopy gaps, accumulation and subsequent removal of coarse woody debris (CWD), and soil disruption caused by wind disturbance followed by salvage logging. Time of disturbance denotes the windthrow event and duration of salvage harvesting (indicated by the bar) denotes the length of time of the salvage logging operation.

Although the severity of salvage logging operations varies based on methods, this practice typically removes or alters the biological legacies created by natural disturbances, which simplifies stand structure, decreases habitat connectivity, alters hydrological properties, influences trajectories of forest recovery, and has cascading effects on local plant and animal communities [10,11]. Intensive and extensive salvage logging is predicted to dynamically alter temporal patterns in the effect sizes of canopy gaps, accumulation and subsequent removal of CWD, and soil properties on ground-dwelling invertebrate communities (Figure 3).

Intensive salvage logging operations typically remove residual living trees that did not fall from wind along with downed trees, increasing the size of canopy gaps. Therefore, the greatest effect of canopy gap formation on invertebrate communities is predicted to occur soon after the logging operation when gaps are at their maximum size. Along with increased light availability reaching the forest floor, complete removal of large trees often compromises essential ecosystem processes [13,122,125,141,142]. The loss of trees and disturbance to the understory decreases transpiration and nutrient uptake and increases leaching, soil and surface moisture levels, and soil temperatures, which can alter nutrient cycling and decomposition processes [4,143,144].

Removal of living and dead trees significantly reduces the amount of CWD in the stand, but also the amount that will be added in the next several decades [12,145,146]. The greatest effect of CWD accumulation is predicted to occur soon after the disturbance and then decrease rapidly during the logging operation until some post-salvaging volume of CWD and FWD remain. Reduction in the volume of downed woody debris leaves forests recovering from salvaging depauperate in the diversity of woody debris size, type, and decay classes characteristic of undisturbed or naturally disturbed forests [147].

The use of heavy machinery and creation of skid trails and roads for tree removal increases compaction and erosion of soil organic and mineral layers [148,149]. The greatest effect of soil disturbance on ground-dwelling invertebrates is predicted to occur soon after salvaging. Physical properties of the soil are altered by increasing bulk density and water run-off, and decreasing structure, aeration, porosity, water infiltration and retention, gas exchange, and root growth [139,140,150,151,152,153]. Increased bulk density ranged from 20–60% [148,154,155] with effects detected 15–60 cm below the soil surface [148,150,156]. Recovery of soils was highly variable with time scales ranging from 5 to ≥40 years [139,150,154,157], likely owing to site-specific soil properties and factors related to the harvesting operation [139,140,154,155].

Salvage logging of downed and residual standing trees following natural disturbance is predicted to have dynamic temporal effects on ground-dwelling invertebrate communities. Effects of CWD accumulation is predicted to be greatest initially after the natural disturbance, while the effects of canopy gap formation, CWD removal, and soil disturbance are predicted to be greatest immediately after salvage logging (Figure 3).

Greenberg and Forrest [39] investigated ground-dwelling invertebrate communities in unsalvaged and salvaged windthrow gaps created by Hurricane Opal and found that activity-abundance and biomass of Thomisidae was lower, while Sclerosomatidae was higher in salvaged gaps. Activity-abundance of Formicidae was higher in salvaged gaps than in unsalvaged windthrow gaps, but biomass remained similar [39], suggesting a reduction in body size in salvaged gaps. Basu [129] found higher Formicidae species richness in salvaged gaps followed by natural tree-fall gaps, and then undisturbed forest, with distinct assemblage composition reported in these three habitats. However, gap size or age was not specified [129], which hinders comparisons between studies and integration into the model framework.

Activity-abundance of total ground-dwelling invertebrates was lower one year and higher two years following intensive salvage logging that mechanically removed downed boles and residual standing trees via skid trails than in unsalvaged forest affected by a tornado [127]. Gastropoda, Scarabaeinae, and Gryllidae were more abundant in salvaged gaps, while Caseyidae, Parajulidae, Entomobryidae, Dicyrtomidae, and Katiannidae were less abundant [127]. Activity-abundances of Spirobolidae, Araneae, Opiliones, Formicidae, and Carabidae were higher, and Scolopendromorpha, Julidae, Geotrupidae, and Aphodiinae were lower in windthrow and salvaged gaps than in undisturbed forest. Distinct invertebrate communities were found after salvaging, whereas composition was more similar in windthrow gaps and undisturbed forest [127], highlighting the importance of downed CWD retention following disturbances that substantially alter the canopy layer.

Urbanovičová et al. [158,159] documented higher activity-abundance of ground-dwelling arthropods largely driven by increased numbers of Collembola and Acari in salvaged spruce forest after windthrow. However, salvaged forest had lower arthropod evenness and diversity, suggesting that these combined disturbances increased the dominance of these two arthropod taxa. Activity-abundances of Carabidae, Curculionidae, Staphylinidae, Scarabaeidae, and Opiliones were higher in undisturbed forest, while Collembola, Acari, Araneae, Chilopoda, and Diplopoda were more abundant in salvaged forest [158,159]. Wermelinger et al. [160] reported higher abundance and species richness of arthropods in unsalvaged and salvaged windthrow sites created by windstorm Lothar. Coleoptera was the exception to this pattern, as higher abundances were observed in undisturbed forest, primarily due to catches of Carabidae [160].

Studies often report increased activity-abundance, richness, and (or) diversity of Carabidae following disturbance due to the colonization of open-habitat and generalist species, but forest species decline resulting in distinct assemblages. Gandhi et al. [53] reported higher species richness and diversity of Carabidae in wind disturbed forests after salvaging than in severely wind disturbed (>70% tree mortality) and undisturbed forest. However, activity-abundances of the forest species *Pterostichus coracinus* (Newman), *Pterostichus pensylvanicus* LeConte, and *Sphaeroderus lecontei* Dejean were higher in undisturbed forest. Phillips et al. [161] reported higher activity-abundance of Carabidae in salvaged forest than in unsalvaged after wildfire, but species diversity was similar owing to changes in species composition. *Pterostichus adstrictus* Eschscholtz, *Sericoda quadripunctata* (DeGeer), and *Sericoda bembidioides* Kirby increased in salvaged forest [161].

Koivula and Spence [162] investigated the effects of low (23–30% timber removed), moderate (40–50%), and high (60–70%) intensity salvage logging after wildfire on Carabidae assemblages at forest stand and landscape scales. Activity-abundance and species richness of Carabidae were higher after salvaging and increased with increasing salvage intensity compared to unsalvaged forest. *Pterostichus punctatissimus* (Randall) increased, whereas *Pterostichus adstrictus* Eschscholtz, *Platynus decentis* (Say), *Calathus ingratus* Dejean, *Agonum retractum* LeConte, *Harpalus laevipes* Zetterstedt, and *Calosoma frigidum* Kirby decreased with increasing salvage intensity [162]. These patterns in Carabidae assemblages were most apparent at landscape scales rather than at the stand-level. Retention of biological legacies in salvaged areas would likely support populations of forest Carabidae species.

Thorn et al. [163] decoupled the effects of canopy gaps and forest floor microhabitats created by windthrow and salvage logging via a small-scale manipulative experiment and found the formation of canopy gaps was the primary driver of Carabidae and Araneae assemblages. They found higher activity-abundance and species richness of epigeal Araneae in canopy gaps, and higher activity-abundance of Carabidae under closed canopy. Canopy gaps also altered species composition of Araneae and Carabidae, but the creation of microhabitats, such as pit-and-mound topography, had minimal impacts [163]. Two carabid species, *Cicindela campestris* Linnaeus and *Bembidion deletum* Audinet-Serville, commonly found in disturbed areas with sparse vegetation, were more abundant in pits. Thorn et al. [163] suggested that the effects of microhabitats created by wind on ground-dwelling invertebrates may emerge at larger spatial scales than investigated in the study.

### 3.4. Responses of Invertebrate Taxa

Effects of canopy gaps, CWD, and salvage logging on ground-dwelling invertebrate communities were highly variable between taxa (Table S1). Moreover, in many studies, effects of disturbance on invertebrates were not detected. Spirobolidae and Formicidae generally responded positively to disturbance, whereas the responses of Scolopendromorpha, Julidae, and Carabidae were generally negative. Ground-dwelling invertebrates were unaffected by, or responded positively to, the presence of CWD, with few taxa negatively impacted. Araneae and Collembola generally responded positively to disturbance when studies analyzed their total abundance, but largely negative responses were documented for individual families of these two taxa. Pseudoscorpiones and Blattodea were consistently unaffected by these disturbances.

When looking at patterns within taxa, highly variable responses were observed for some ground-dwelling invertebrates. For example, responses of Opiliones and families of Coleoptera (excluding Carabidae) were inconsistent for canopy gap formation, patterns of CWD accumulation and removal, and soil disturbance. Responses of Carabidae were largely consistent among studies for canopy gaps created by invasive insect-induced tree mortality, but more variable following salvage logging, perhaps due to variation in site-specific factors and differences in the harvesting operations between studies.

Studies that use community metrics, such as total activity-abundance, taxonomic richness, evenness, and diversity found varying responses of ground-dwelling invertebrates to large canopy disturbance caused by wind and salvage logging, but largely consistent responses to small canopy gaps created by invasive insect-induced tree mortality. Canopy gaps created by invasive insects that kill trees tend to be similar in size to those formed by gap-phase dynamics, although there are other temporal and spatial factors that distinguish these two phenomena. Ground-dwelling invertebrates may be well adapted to environmental changes that occur following small-scale canopy gaps, regardless of the disturbance agent. For example, Carabidae assemblages were resilient to canopy gaps created by EAB-induced ash mortality [40,92]. Ground-dwelling invertebrate responses to other disturbance agents including wind and salvage logging were more variable, perhaps due to differences in disturbance properties, forest site conditions, gap characteristics, or numerous other factors that vary with, or independently of, the disturbance event. No effects were detected in many of the studies reviewed, but when effects were reported, ground-dwelling invertebrates tended to respond negatively to larger canopy gaps and positively to the presence of CWD.

Responses of some invertebrate taxa were more commonly evaluated after disturbance than others. Studies that investigated the effects of natural and anthropogenic disturbances on ground-dwelling invertebrate communities focused largely on Diplopoda, Araneae, Formicidae, and Coleoptera, including Carabidae and Staphylinidae. Some arthropods, such as Araneae, Carabidae, and Formicidae, are widely used as focal taxa because they are considered biological indicators, with species highly responsive to changes in habitat or microclimate factors on the forest floor [77,78,80]. Annelida, Gastropoda, Diplura, Blattodea, and Diptera were underrepresented in the studies reviewed, perhaps because of the sampling methods.

## 4. Assessment of Conceptual Models and Conclusions

Three conceptual models described here make testable predictions regarding the temporal relationships in the effect sizes of canopy gap formation, accumulation (and removal) of CWD, and soil disruption for disturbances caused by invasive insect-induced tree mortality, wind storms, and salvage logging following natural disturbance. Some predictions are better supported than others, and these models can provide a basis for structuring future research. Responses of ground-dwelling invertebrates to EAB-induced ash mortality supported the first conceptual model outlining an inverse temporal relationship in the effects of canopy gaps and CWD accumulation, but responses to tree mortality caused by other invasive insects were inconsistent and understudied. Predicted short-term effects of canopy gaps and CWD caused by wind storms and salvage logging outlined in the second and third conceptual models were generally supported, but more studies are required to evaluate predictions of long-term impacts on ground-dwelling invertebrate communities. These models provide a framework to synthesize existing studies and enlighten the design of future experiments.

This review highlights knowledge gaps in understanding the temporal effects of natural and anthropogenic disturbances on ground-dwelling invertebrate communities. Most studies have investigated the initial short-term effects of canopy gap formation, CWD accumulation, and salvage logging practices, but long-term studies are under-represented in the literature. A meta-analysis conducted by Thorn et al. [164] found that most studies assessed the responses of biodiversity less than five years after salvage logging. However, Sklodowski and Garbalinska [131] reported Carabidae assemblages had not recovered six years after a tornado, which suggests studies must monitor communities on longer time scales to assess recovery. Future research should aim to address long-term impacts of disturbance on ground-dwelling invertebrate communities to fill this knowledge gap. Moreover, effects of soil disturbance following salvage logging were not quantitatively assessed in most studies, confounding the impacts with those of decreased volume of CWD. Combined effects of canopy and ground-level factors that occur following natural and anthropogenic disturbances must be decoupled experimentally to improve understanding of their individual effects on forest biodiversity over time.

## Supplementary Materials

The following are available online at https://www.mdpi.com/2075-4450/10/3/61/s1. Table S1: Summary of studies investigating the effects of canopy gap formation, coarse woody debris (CWD) accumulation, and salvage harvesting in forest ecosystems.
