# Supplementary material for: Dynamic Responses of Ground-Dwelling Invertebrate Communities to Disturbance in Forest Ecosystems
Source: Insects. 2019 Feb 26;10(3):61. doi: 10.3390/insects10030061 (PMC6468525; doi:10.3390/insects10030061)
Supplement: Supplementary file 1 [file insects-10-00061-s001.pdf]

Perry, KI and DA Herms. Dynamic responses of ground-dwelling invertebrate communities to disturbance in forest ecosystems, *Insects*

Supplementary Materials

Table S1. Summary of studies investigating the effects of canopy gap formation, coarse woody debris (CWD) accumulation, and salvage harvesting in forest ecosystems. Response variables of ground-dwelling invertebrates were abundance (A), richness (R), evenness (E), diversity (D), and biomass (B). Effects of canopy gaps, CWD, and salvage logging on ground-dwelling invertebrates are represented as positive (+), negative (–), or no effect (≈) ($\alpha \leq 0.05$); the absence of a symbol indicates the factor was not directly investigated in the study.

**Taxon**	**Order, Family, and/or Subfamily**	**Response Measured**	**Disturbance Type**		**Study Location**	**Canopy**	**CWD**	**Salvage**	**Reference**
Annelida		A	Insect	Emerald ash borer	Michigan		+		Ulyshen et al. [101]
		A	Wind	Tornado, Salvage	Pennsylvania	≈	≈	≈	Perry [127]
Gastropoda		A	Insect	Emerald ash borer	Ohio		+		Perry and Herms [50]
		A	Insect	Emerald ash borer	Michigan	≈	≈		Perry and Herms [98]
		A	Wind	Tornado, Salvage	Pennsylvania	≈	≈	+	Perry [127]
Chilopoda		A	Insect	Emerald ash borer	Michigan	≈	≈		Ulyshen et al. [101]
		A	Insect	Emerald ash borer	Ohio	≈	≈		Perry and Herms [50]
		A	Wind, Human	Windstorm, Salvage	Slovakia	+	≈	+	Urbanovičová et al. [158]
	Scolopendromorpha	A	Wind	Tornado, Salvage	Pennsylvania	–	–		Perry [127]
	Scolopendromorpha	A, B	Wind, Human	Hurricane, Salvage	North Carolina	–			Greenberg and Forrest [39]
	Lithobiomorpha	A	Wind	Tornado, Salvage	Pennsylvania	≈	≈	≈	Perry [127]
Diplopoda		A	Insect	Emerald ash borer	Michigan		+		Ulyshen et al. [101]
		A	Wind	Hurricane	Puerto Rico	–	≈		Richardson et al. [41]
		A	Wind, Human	Windstorm, Salvage	Slovakia	+	≈	+	Urbanovičová et al. [158]
	Chordeumatida, Caseyidae	A	Wind	Tornado, Salvage	Pennsylvania	≈	≈	–	Perry [127]
	Julida	A, B	Wind, Human	Hurricane, Salvage	North Carolina	–			Greenberg and Forrest [39]
	Julida, Julidae	A	Wind	Tornado, Salvage	Pennsylvania	–	–	–	Perry [127]
	Julida, Parajulidae	A	Wind	Tornado, Salvage	Pennsylvania	≈	≈	–	Perry [127]
	Polydesmida	A	Insect	Emerald ash borer	Ohio	+			Perry and Herms [50]
	Polydesmida	A	Insect	Emerald ash borer	Michigan	≈	≈		Perry and Herms [98]
	Polydesmida	A, B	Wind, Human	Hurricane, Salvage	North Carolina	≈	≈	≈	Greenberg and Forrest [39]
	Polydesmida, Paradoxosomatidae	A	Wind	Tornado, Salvage	Pennsylvania	≈	≈	≈	Perry [127]
	Polydesmida, Polydesmidae	A	Wind	Tornado, Salvage	Pennsylvania	≈	≈	≈	Perry [127]
	Spirobolida	A, B	Wind, Human	Hurricane, Salvage	North Carolina	–			Greenberg and Forrest [39]
	Spirobolida, Spirobolidae	A	Insect	Emerald ash borer	Michigan	≈	+		Perry and Herms [98]
	Spirobolida, Spirobolidae	A	Wind	Tornado, Salvage	Pennsylvania	+	+	+	Perry [127]
Isopoda		A	Insect	Emerald ash borer	Ohio		+		Perry and Herms [50]
		A	Insect	Emerald ash borer	Michigan		+		Ulyshen et al. [101]
		A	Insect	Emerald ash borer	Michigan	≈	+		Perry and Herms [98]
		A	Wind	Hurricane	Puerto Rico	–	≈		Richardson et al. [41]
		A	Wind	Tornado, Salvage	Pennsylvania	≈	≈	≈	Perry [127]
		A	Wind, Human	Windstorm, Salvage	Slovakia	≈	≈	≈	Urbanovičová et al. [158]
Acari		A	Insect	Emerald ash borer	Michigan	≈	≈		Ulyshen et al. [101]
		A	Wind	Hurricane	Puerto Rico	+	≈		Richardson et al. [41]
		A, B	Wind, Human	Hurricane, Salvage	North Carolina	≈	≈	≈	Greenberg and Forrest [39]
		A	Wind, Human	Windstorm, Salvage	Slovakia	+	≈	+	Urbanovičová et al. [158]
Araneae		A	Insect	Emerald ash borer	Michigan		+		Ulyshen et al. [101]
		A	Insect	Emerald ash borer	Ohio	≈	≈		Perry and Herms [50]
		A	Insect	Emerald ash borer	Michigan	≈	≈		Perry and Herms [98]

		A	Wind	Tornado, Salvage	Pennsylvania	+	+	+	Perry [127]
		A, R	Wind, Human	Windstorm, Salvage	Switzerland	+	+	+	Wermelinger et al. [160]
		D	Wind, Human	Windstorm, Salvage	Switzerland	+	–	+	Wermelinger et al. [160]
		A	Wind, Human	Windstorm, Salvage	Slovakia	+	≈	+	Urbanovičová et al. [158]
		A	Human	Experimental, Emulate HWA	Massachusetts	+	≈	≈	Sackett et al. [105]
		R	Human	Experimental, Emulate HWA	Massachusetts	+	≈	≈	Sackett et al. [105]
		A, R	Human	Experimental, Salvage	Germany	+	≈	≈	Thorn et al. [163]
	Atypidae	A, B	Wind, Human	Hurricane, Salvage	North Carolina	–			Greenberg and Forrest [39]
	Gnaphosidae	A, B	Wind, Human	Hurricane, Salvage	North Carolina	≈	≈	≈	Greenberg and Forrest [39]
	Lycosidae	A, B	Wind, Human	Hurricane, Salvage	North Carolina	≈	≈	≈	Greenberg and Forrest [39]
	Pisauridae	A, B	Wind, Human	Hurricane, Salvage	North Carolina	–			Greenberg and Forrest [39]
	Thomisidae	A, B	Wind, Human	Hurricane, Salvage	North Carolina	–			Greenberg and Forrest [39]
Opiliones		A	Insect	Emerald ash borer	Ohio	–			Perry and Herms [50]
		A	Insect	Emerald ash borer	Michigan	≈	–		Perry and Herms [98]
		A	Insect	Emerald ash borer	Michigan		+		Ulyshen et al. [101]
		A	Wind	Tornado, Salvage	Pennsylvania	+	+	+	Perry [127]
		A	Wind, Human	Windstorm, Salvage	Slovakia	–	≈	–	Urbanovičová et al. [158]
		A, R	Human	Experimental, Salvage	Germany	≈	≈	≈	Thorn et al. [163]
	Sclerosomatidae	A, B	Wind, Human	Hurricane, Salvage	North Carolina	+		+	Greenberg and Forrest [39]
Pseudoscorpiones		A	Insect	Emerald ash borer	Michigan	≈	≈		Ulyshen et al. [101]
		A	Wind	Tornado, Salvage	Pennsylvania	≈	≈	≈	Perry [127]
		A	Wind, Human	Windstorm, Salvage	Slovakia	≈	≈	≈	Urbanovičová et al. [158]
Diplura		A	Wind, Human	Windstorm, Salvage	Slovakia	≈	≈	≈	Urbanovičová et al. [158]
Collembola		A	Insect	Emerald ash borer	Michigan		+		Ulyshen et al. [101]
		A	Insect	Emerald ash borer	Michigan	≈	+		Perry and Herms [98]
		A	Wind	Hurricane	Puerto Rico	+			Richardson et al. [41]
		A	Wind, Human	Windstorm, Salvage	Slovakia	+	≈	+	Urbanovičová et al. [158]
	Onychiuridae	A	Wind	Tornado, Salvage	Pennsylvania	≈	≈	≈	Perry [127]
	Hypogastruridae	A	Insect	Emerald ash borer	Ohio	–			Perry and Herms [50]
	Hypogastruridae	A	Wind	Tornado, Salvage	Pennsylvania	≈	≈	≈	Perry [127]
	Isotomidae	A	Insect	Emerald ash borer	Ohio	–			Perry and Herms [50]
	Isotomidae	A	Wind	Tornado, Salvage	Pennsylvania	≈	≈	≈	Perry [127]
	Sminthuridae	A	Insect	Emerald ash borer	Ohio	–			Perry and Herms [50]
	Tomoceridae	A	Insect	Emerald ash borer	Ohio	≈	≈		Perry and Herms [50]
	Tomoceridae	A	Wind	Tornado, Salvage	Pennsylvania	≈	≈	≈	Perry [127]
	Entomobryidae	A	Insect	Emerald ash borer	Ohio	≈	≈		Perry and Herms [50]
	Entomobryidae	A	Wind, Human	Tornado, Salvage	Pennsylvania	≈	≈	–	Perry [127]
	Dicyrtomidae	A	Wind, Human	Tornado, Salvage	Pennsylvania	≈	≈	–	Perry [127]
	Katiannidae	A	Wind, Human	Tornado, Salvage	Pennsylvania	≈	≈	–	Perry [127]
	Neanuridae	A	Wind, Human	Tornado, Salvage	Pennsylvania	≈	≈	≈	Perry [127]
Coleoptera		A	Insect	Emerald ash borer	Michigan		+		Ulyshen et al. [101]
		R	Insect	Emerald ash borer	Michigan		+		Ulyshen et al. [101]
		A	Wind, Human	Windstorm, Salvage	Switzerland	≈	≈	≈	Wermelinger et al. [160]
		R	Wind, Human	Windstorm, Salvage	Switzerland	+	+	+	Wermelinger et al. [160]
		D	Wind, Human	Windstorm, Salvage	Switzerland	+	≈	+	Wermelinger et al. [160]
		A	Human	Experimental, Emulate HWA	Massachusetts	–	≈	≈	Sackett et al. [105]
		R	Human	Experimental, Emulate HWA	Massachusetts	≈	≈	≈	Sackett et al. [105]
	Carabidae	A, R, D	Insect	Emerald ash borer	Michigan	–			Gandhi et al. [92]
	Carabidae	A	Insect	Emerald ash borer	Ohio	–			Perry and Herms [50]
	Carabidae	A	Insect	Emerald ash borer	Ohio	–			Perry and Herms [40]
	Carabidae	A	Insect	Emerald ash borer	Michigan	≈	≈		Perry and Herms [98]
	Carabidae	A, R,	Wind	Tornado	Illinois	≈	≈		Barber and Widick [132]
	Carabidae	D, C	Wind	Tornado	Illinois	+	+		Barber and Widick [132]
	Carabidae	A	Wind	Hurricane	Poland	–	–		Skłodowski and Garbalińska [134]

	Carabidae	R	Wind	Hurricane	Poland	+	+		Skłodowski and Garbalińska [134]
	Carabidae	R	Wind	Tornado	Poland	+	+		Sklodowski and Garbalinska [131]
	Carabidae	A	Wind, Human	Windstorm, Salvage	Minnesota	–	–	–	Gandhi et al. [53]
	Carabidae	R, D	Wind, Human	Windstorm, Salvage	Minnesota	+	+	+	Gandhi et al. [53]
	Carabidae	A	Wind, Human	Tornado, Salvage	Pennsylvania	+	+	+	Perry [127]
	Carabidae	A	Wind, Human	Windstorm, Salvage	Slovakia	–	≈	–	Urbanovičová et al. [158]
	Carabidae	A	Wind, Human	Hurricane, Salvage	North Carolina	–			Greenberg and Forrest [39]
	Carabidae	B	Wind, Human	Hurricane, Salvage	North Carolina	+	≈	≈	Greenberg and Forrest [39]
	Carabidae	A	Human	Experimental, Salvage	Germany	≈	≈	≈	Thorn et al. [163]
	Carabidae	R	Human	Experimental, Salvage	Germany	–	≈	≈	Thorn et al. [163]
	Carabidae	A, R	Fire, Human	Wildfire, Salvage	Canada	+		+	Koivula and Spence [162]
	Carabidae	A	Fire, Human	Wildfire, Salvage	Canada			+	Phillips et al. [161]
	Carabidae	D	Fire, Human	Wildfire, Salvage	Canada			≈	Phillips et al. [161]
	Curculionidae	A	Wind, Human	Tornado, Salvage	Pennsylvania	≈	≈	≈	Perry [127]
	Curculionidae	A, B	Wind, Human	Hurricane, Salvage	North Carolina	≈	≈	≈	Greenberg and Forrest [39]
	Curculionidae	A	Wind, Human	Windstorm, Salvage	Slovakia	–	≈	–	Urbanovičová et al. [158]
	Curculionidae, Scolytinae	A	Wind	Tornado, Salvage	Pennsylvania	+	+	≈	Perry [127]
	Elateridae	A, B	Wind, Human	Hurricane, Salvage	North Carolina	≈	≈	≈	Greenberg and Forrest [39]
	Geotrupidae	A	Wind	Tornado, Salvage	Pennsylvania	–	–	–	Perry [127]
	Histeridae	A	Wind	Tornado, Salvage	Pennsylvania	≈	≈	≈	Perry [127]
	Histeridae	A, B	Wind, Human	Hurricane, Salvage	North Carolina	≈	≈	≈	Greenberg and Forrest [39]
	Nitidulidae	A	Insect	Emerald ash borer	Ohio	≈	≈		Perry and Herms [50]
	Nitidulidae	A	Insect	Emerald ash borer	Michigan	≈	≈		Perry and Herms [98]
	Nitidulidae	A	Wind	Tornado, Salvage	Pennsylvania	≈	≈	≈	Perry [127]
	Phalacrididae	A	Wind	Tornado, Salvage	Pennsylvania	≈	+	≈	Perry [127]
	Ptillidae	A	Wind	Tornado, Salvage	Pennsylvania	≈	+	≈	Perry [127]
	Scarabaeidae	A	Insect	Emerald ash borer	Ohio	–			Perry and Herms [50]
	Scarabaeidae	A	Insect	Emerald ash borer	Michigan	≈	≈		Perry and Herms [98]
	Scarabaeidae	A	Wind	Tornado, Salvage	Pennsylvania	≈	≈	≈	Perry [127]
	Scarabaeidae	A, B	Wind, Human	Hurricane, Salvage	North Carolina	≈	≈	≈	Greenberg and Forrest [39]
	Scarabaeidae	A	Wind, Human	Windstorm, Salvage	Slovakia	–	≈	–	Urbanovičová et al. [158]
	Scarabaeidae, Aphodinae	A	Wind	Tornado, Salvage	Pennsylvania	–	–	–	Perry [127]
	Scarabaeidae, Scarabaeinae	A	Wind	Tornado, Salvage	Pennsylvania	≈	≈	+	Perry [127]
	Silphidae	A	Insect	Emerald ash borer	Ohio	≈	≈		Perry and Herms [50]
	Silphidae	A	Insect	Emerald ash borer	Michigan	≈	≈		Perry and Herms [98]
	Silphidae	A	Wind	Tornado, Salvage	Pennsylvania	≈	≈	≈	Perry [127]
	Silphidae	A, B	Wind, Human	Hurricane, Salvage	North Carolina	≈	≈	≈	Greenberg and Forrest [39]
	Staphylinidae	A	Insect	Emerald ash borer	Ohio	≈	≈		Perry and Herms [50]
	Staphylinidae	A	Insect	Emerald ash borer	Michigan	≈	≈		Perry and Herms [98]
	Staphylinidae	A	Wind	Tornado, Salvage	Pennsylvania	≈	+	≈	Perry [127]
	Staphylinidae	A, B	Wind, Human	Hurricane, Salvage	North Carolina	≈	≈	≈	Greenberg and Forrest [39]
	Staphylinidae	A	Wind, Human	Windstorm, Salvage	Slovakia	–	≈	–	Urbanovičová et al. [158]
	Tenebrionidae	A, B	Wind, Human	Hurricane, Salvage	North Carolina	≈	≈	≈	Greenberg and Forrest [39]
Diptera		A	Insect	Emerald ash borer	Michigan	≈	≈		Ulyshen et al. [101]
Hymenoptera		A	Wind, Human	Windstorm, Salvage	Slovakia	≈	≈	≈	Urbanovičová et al. [158]
	Formicidae	A	Insect	Emerald ash borer	Michigan	≈	≈		Ulyshen et al. [101]
	Formicidae	A	Insect	Emerald ash borer	Ohio	≈	≈		Perry and Herms [50]
	Formicidae	A	Insect	Emerald ash borer	Michigan	≈	≈		Perry and Herms [98]
	Formicidae	A, R	Insect	Hemlock woolly adelgid	Massachusetts	+		+	Kendrick et al. [103]
	Formicidae	A	Wind	Tornado, Salvage	Pennsylvania	+	+	+	Perry [127]
	Formicidae	A	Wind	Hurricane	Puerto Rico	≈	≈		Richardson et al. [41]
	Formicidae	R	Wind	Storms, Temporales	Costa Rica	+			Patrick et al. [128]
	Formicidae	A, B	Wind, Human	Hurricane, Salvage	North Carolina	≈	≈	≈	Greenberg and Forrest [39]
	Formicidae	A	Human	Experimental, Emulate HWA	Massachusetts	+	≈	≈	Sackett et al. [105]
	Formicidae	R	Human	Experimental, Emulate HWA	Massachusetts	+	≈	≈	Sackett et al. [105]
	Formicidae	E, D	Human	Experimental, Emulate HWA	Massachusetts	+			Record et al. [106]
	Formicidae	A, R	NA	Canopy Gap	Panama	≈			Feener and Schupp [130]

	Formicidae	A, R	NA, Human	NA, Salvage	India	+		+	Basu [129]
Hemiptera		A	Insect	Emerald ash borer	Michigan	≈	≈		Ulyshen et al. [101]
		A	Wind	Hurricane	Puerto Rico	–			Richardson et al. [41]
		A, B	Wind, Human	Hurricane, Salvage	North Carolina	≈	≈	≈	Greenberg and Forrest [39]
		A, R, D	Wind, Human	Windstorm, Salvage	Switzerland	+	≈	+	Wermelinger et al. [160]
Blattodea	Blattellidae	A, B	Wind, Human	Hurricane, Salvage	North Carolina	≈	≈	≈	Greenberg and Forrest [39]
	Cryptoceridae	A, B	Wind, Human	Hurricane, Salvage	North Carolina	≈	≈	≈	Greenberg and Forrest [39]
Orthoptera	Acrididae	A, B	Wind, Human	Hurricane, Salvage	North Carolina	≈	≈	≈	Greenberg and Forrest [39]
	Gryllidae	A	Insect	Emerald ash borer	Ohio	≈	≈		Perry and Herms [50]
	Gryllidae	A	Insect	Emerald ash borer	Michigan	≈	≈		Perry and Herms [98]
	Gryllidae	A	Wind	Tornado, Salvage	Pennsylvania	+	≈	+	Perry [127]
	Gryllidae	A, B	Wind, Human	Hurricane, Salvage	North Carolina			+	Greenberg and Forrest [39]
	Gryllacrididae	A, B	Wind, Human	Hurricane, Salvage	North Carolina	≈	≈	≈	Greenberg and Forrest [39]
	Rhaphidophoridae	A	Insect	Emerald ash borer	Ohio	–			Perry and Herms [50]
	Rhaphidophoridae	A	Insect	Emerald ash borer	Michigan	≈	≈		Perry and Herms [98]
	Rhaphidophoridae	A	Wind	Tornado, Salvage	Pennsylvania	≈	≈	≈	Perry [127]
Psocoptera		A	Insect	Emerald ash borer	Michigan	+			Ulyshen et al. [101]
		A	Wind	Hurricane	Puerto Rico	+			Richardson et al. [41]
		A	Wind, Human	Windstorm, Salvage	Slovakia	≈	≈	≈	Urbanovičová et al. [158]
Community		R, D	Insect	Emerald ash borer	Ohio	–			Perry and Herms [50]
		A	Insect	Emerald ash borer	Ohio	≈	≈		Perry and Herms [50]
		A, E, D	Insect	Emerald ash borer	Michigan	≈	≈		Perry and Herms [98]
		R	Insect	Emerald ash borer	Michigan	≈	+		Perry and Herms [98]
		R,E	Insect	Beech Bark Disease	New York, Vermont	≈			Garneau et al. [112]
		A	Wind	Tornado, Salvage	Pennsylvania	+	+	+	Perry [127]
		R, E, D	Wind	Tornado, Salvage	Pennsylvania	+	+	≈	Perry [127]
		A	Wind	Hurricane	Puerto Rico	≈	≈		Richardson et al. [41]
		B, D	Wind	Hurricane	Puerto Rico	–			Richardson et al. [41]
		A, B	Wind, Human	Hurricane, Salvage	North Carolina	–			Greenberg and Forrest [39]
		A	Wind, Human	Windstorm, Salvage	Slovakia	+	≈	+	Urbanovičová et al. [158]
		E, D	Wind, Human	Windstorm, Salvage	Slovakia	–	≈	–	Urbanovičová et al. [158]
